# The use of virtual reality during medical procedures in a pediatric orthopedic setting: A mixed‐methods pilot feasibility study

**DOI:** 10.1002/pne2.12078

**Published:** 2022-04-14

**Authors:** Sofia Addab, Reggie Hamdy, Sylvie Le May, Kelly Thorstad, Argerie Tsimicalis

**Affiliations:** ^1^ McGill University Montreal QC Canada; ^2^ Shriners Hospitals for Children®‐Canada Montreal QC Canada; ^3^ CHU Sainte‐Justine Montreal QC Canada; ^4^ Université de Montréal Montreal QC Canada

**Keywords:** adolescents, children, distraction, musculoskeletal conditions, procedural anxiety, procedural pain, virtual reality

## Abstract

Medical procedures cause pain and anxiety in children. Distraction techniques, including virtual reality (VR), may be used in healthcare settings to reduce rates of undertreated procedural pain and anxiety. A mixed‐methods, concurrent triangulation design was piloted at a pediatric orthopedic hospital to assess the feasibility, clinical utility, tolerability, and initial clinical efficacy of VR distraction during medical procedures received by patients with complex musculoskeletal conditions. Questionnaire, scale, interview, observation, and focus group data were collected from patients, their parents, and healthcare professionals. Triangulation of key quantitative and qualitative findings produced final themes and meta‐themes. A total of 44 patients and their parents undergoing intravenous insertions (n = 30), pin removals (n = 7), blood draws (n = 3), Botox injections (n = 2), dressing change (n = 1), and urodynamic test (n = 1) were recruited along with 11 healthcare professionals performing the medical procedures. The following themes resulted from triangulation of data sources: VR intervention was (a) feasible because VR was easily implemented into the clinical workflow, (b) clinically useful as VR was accepted by stakeholders and easy to use, (c) tolerable as VR caused minimal discomfort, and (d) showed initial clinical efficacy in managing procedural pain and anxiety. These findings will inform policies and procedures for VR use in practice and a sustainable implementation across the [name of hospital removed for peer review] network.

## 1 INTRODUCTION

### 1.1 Painful and anxiety‐inducing medical procedures in children

Medical procedures, such as intravenous insertions and percutaneous pin removals, cause pain and anxiety in children.^1^, ^2^, ^3^, ^4^ Although preventable, children continue to report high rates of procedural pain in hospital settings.^1^ A plethora of evidence for effective pharmacological and nonpharmacological pain interventions exists; however, these interventions are underutilized in the clinical setting.^5^, ^6^ Undertreated procedural pain is associated with anxiety and fear for future medical procedures.^6^ Further, recalls of painful or anxiety‐inducing experiences may lead to patient and family dissatisfaction, emotional stress, and delayed healing.^5^ The state of anxiety that accompanies painful procedures and the negative memories formed during a first painful experience lead to traumatic recalls and exaggerated painful memories.^7^, ^8^ A cyclic effect ensues with heightened fear and pain perception during future medical procedures, and avoidance of future healthcare encounters persisting well into adulthood.^7^

Children with chronic conditions, who undergo repeated medical procedures as part of their standard care, remain at heightened risk of undertreated, procedural pain and anxiety.^9^ A study comparing the pain perceived during venipuncture in children with and without a chronic condition showed that children with a chronic condition experienced more pain and displayed more signs of behavioral distress.^9^ These findings challenge some healthcare professionals who believe that repeated pain exposures lead to increased pain tolerance and lower pain perception.^9^ Such misconceptions about pediatric procedural pain experiences and the memory of pain impacting future pain experiences^7^, ^8^ highlight the need for providing feasible and clinically effective pain interventions during routine medical procedures in the care of chronic illnesses.

### 1.2 Virtual reality as a distraction tool

Distraction is a cognitive intervention with empirical evidence supporting its use for procedural pain and anxiety management.^4^ Distraction avoids the undesirable side effects and costs of pharmacological interventions while offering a humanistic approach to care. This cognitive tool works by shifting one's attention away from unpleasant stimuli to more enjoyable ones. In the context of medical procedures, distraction tools take the attention away from the procedure and hospital setting, reducing the perception of pain and anxiety. Reviews support the efficacy of various distraction interventions for procedural pain and distress in children and adolescents,^4^ including the use of virtual reality (VR).^10^

Virtual reality is a powerful and novel form of active and immersive distraction that has emerged in the healthcare setting. Through a VR headset that conveniently blocks cues from the physical environment, the user enters a computer‐generated, three‐dimensional virtual world. During a medical procedure, engaging in a VR experience diverts patients' attention away from painful stimuli and immerses them in a pleasant virtual world, thereby decreasing their pain perception.^11^ Hoffman and colleagues pioneered the use of VR distraction during burn wound care procedures with children.^12^, ^13^ Later, VR distraction use expanded to other painful and anxiety‐inducing procedures received by pediatric patients, including postburn physiotherapy, dental, cancer‐related, needle‐related, and perioperative procedures.^10^, ^14^, ^15^, ^16^ Considering the well‐documented link between pain and anxiety, VR has been leveraged as an anxiolytic tool with children, which may also be used during medical procedures.^10^, ^14^, ^15^

Despite the growing body of research and evidence for the use of VR distraction during painful and anxiety‐inducing medical procedures in children, a gap remains in the use of VR in children with complex musculoskeletal conditions, who undergo routine and repeated medical procedures as part of their long‐term care. Before the long‐term implementation of VR in clinical practice, a feasibility trial must be piloted to ensure key stakeholders will accept and use the distraction tool. The study purpose was to investigate the feasibility, clinical utility, tolerability, and initial clinical efficacy of using VR with children with complex musculoskeletal conditions undergoing a medical procedure.

## 2 METHODS

### 2.1 Study design and setting

Following ethical approval from the institutional review board (A06‐M31‐19B), a mixed‐method, concurrent triangulation feasibility study was piloted at a university‐affiliated, not‐for‐profit, pediatric orthopedic hospital located in [location removed for peer review]. The study was guided by the Virtual Reality Clinical Outcomes Research Experts (VR‐CORE) methodological framework, a three‐part methodological framework for the design and testing of VR studies in healthcare.^17^ The present study followed the VR2 trial design, wherein the VR intervention selected undergoes early testing. This trial is guided by six best‐practice recommendations for testing VR in (1) the intended clinical setting and (2) targeted population; and examining the following VR‐specific clinical outcomes (3) feasibility, (4) acceptability, (5) tolerability, and (6) initial clinical efficacy.^17^ The selected mixed‐method design permitted the concurrent, one‐phase collection and analysis of different quantitative and qualitative data sources.^18^

Feasibility was defined by the barriers and facilitators in using VR in the clinical settings of the study site setting and the time required to administer the VR intervention. Clinical utility was defined as the acceptability, ease of use, ease of understanding, satisfaction, and recommendation of the VR intervention from the perspective of all stakeholders. Tolerability was defined as the absence of physical or emotional adverse effects and the absence of perceived discomfort or inconvenience from the VR intervention. Finally, initial clinical efficacy was defined by two main clinically relevant and validated patient‐reported outcomes of the VR intervention: procedural pain and anxiety. Both quantitative and qualitative methods were used to evaluate all outcomes.

### 2.2 Participants

Convenience sampling techniques were used to prospectively recruit participants working (n = 8‐12), or seeking healthcare (n = 44), in the varying clinics at the study site. Participants included key stakeholders in VR use: children and adolescents, their accompanying parents/caregivers, and healthcare professionals (HCPs) administering medical care. Children and adolescents were included in the study if they: (1) were between the ages of 5 and 21 years old, (2) had a scheduled medical procedure (i.e., pin removals, cast changes, dressing changes, pressure ulcer debridement, intravenous insertions, blood draws, staples removal, installation of traction, or urodynamic test), and (3) were fluent in French or English. Children and adolescents were excluded whether they had a cognitive, auditory, or visual impairment preventing them from using VR, had an epilepsy diagnosis, or whether they were unable to sit semi‐upright during their medical procedure. Healthcare professionals were included whether they performed or supported the patient undergoing a medical procedure. Not all parents remain present during medical procedures; thus, parent participation was not mandatory, but those parents who were present were invited to share their perspective on VR during interviews.

The sample size estimate (range = 10‐40) was justified based on the VR‐CORE recommendation to recruit “a large enough sample to represent the breadth and depth of target patients and provide statistically stable estimates in descriptive analytics.”^17^ Hence, participants up to the age of 21 and receiving a wide range of procedures were recruited to represent the study site's patient population and medical procedures offered. Medical procedures included in the study were selected based on a focus group conducted with healthcare managers and professionals at the study site, prioritizing procedures that anecdotally caused the most pain or anxiety to patients, including IV insertions, pin/wire removals, cast changes, blood draws, Botox injections, and urodynamics tests.

### 2.3 Study procedures and data collection

Healthcare professionals helped identify potentially eligible participants according to a printed study information sheet provided by the research team. With the families' permission, a member of the research team explained the study and, whether agreeable, obtained informed consent and/or assent. Baseline sociodemographic and clinical information were subsequently collected using hospital charts and patient/parent reports. Baseline patient‐reported pain, anxiety, and simulator sickness were then collected using the FACES Pain Scale–Revised (FPS‐R),^19^ FACES Anxiety Scale (FAS) for children,^20^ and Child Simulator Sickness Questionnaire (CSSQ),^21^ respectively.

The VR intervention was explained, and the headset was fitted to the child, ensuring they could clearly see the depicted VR world. Each child had about 5 minutes of immersive play before the HCP started the medical procedure (conducted as per standard hospital protocol). Throughout the VR intervention, the researcher remained on stand‐by for the child and HCP, answered any questions, troubleshooted, and recorded the observations and fieldnotes. When possible, direct quotes were transcribed verbatim. Patients played the VR game for the duration of the medical procedure unless they voluntarily removed the VR headset or requested to stop the VR intervention.

Following the medical procedure, the VR headset was removed, and the equipment was disinfected after each use. Postintervention outcomes were then collected immediately after the medical procedure and included the same baseline outcome measures (i.e., the FPS‐R, FAS, CSSQ) followed by a Graphic Rating Scale (GRS) evaluating multidimensional pain,^23^ and Patient Perception Questionnaire.^22^ Patients and their parents (if present) then participated in short, audio‐recorded semi‐structured interviews. Healthcare professionals filled out a perception questionnaire^22^ after each medical procedure conducted with the use of VR and debriefed with the researcher. A mid‐study focus group discussion was conducted with the HCPs to review their experience, questions, or concerns with the use of VR in their clinic. Notes from the discussions were summarized with the help of transcribed audio recordings.

### 2.4 Instruments

The following instruments were completed.

#### 2.4.1 Patient sociodemographic and clinical questionnaire

Patients' age, sex, date of birth, race/ethnicity, medical diagnosis, and scheduled medical procedure were collected. Pain and anxiety medications taken prior to the medical procedure or as part of the standard procedures were noted.

#### 2.4.2 FACES pain scale–revised

The FACES Pain Scale–Revised is a 1‐item self‐report measure of pain intensity that may be used in children aged four and above.^19^ The scale has six faces placed on a scoring metric (0‐10), showing different levels of pain intensity ranging from someone that feels “no pain” (0) to someone that feels “very much pain” (10).

#### 2.4.3 The FACES anxiety scale (FAS) for children

The FACES Anxiety Scale for Children is a 1‐item self‐report measure of state anxiety^20^ scored from 0 to 4. The scale shows five faces with increasing levels of anxiety, with the first face showing no anxiety (a score of 0) and the last face showing extreme anxiety (a score of 4).

#### 2.4.4 Graphic rating scale for multidimensional pain assessment

The Graphic Rating Scale (GRS)^23^ is a self‐report measure of fun, nausea, and three subjective pain dimensions consisting of cognitive (“time spent thinking about pain”), affective (“pain unpleasantness”), and sensory (“worst pain”). Each item is rated on a 10 cm line, with a scoring metric ranging from 0 to 10. Along the line, descriptive markers are added, such as “mild,” “moderate,” and “severe” to help participants make the appropriate self‐assessment.

#### 2.4.5 Child simulator sickness questionnaire

A 7‐item questionnaire developed by Hoeft et al. (2003) was used to assess the presence of cybersickness. The items are grouped into three symptom categories: (a) nausea, (b) oculomotor, and (c) disorientation. A score of three or greater, in any one of the three categories, signals the presence of simulator sickness.

#### 2.4.6 Patient perception questionnaire

A 5‐item questionnaire modified from Ford et al. (2018) was used to assess patients' perception and satisfaction with the VR intervention by replacing words specific to burn wound care with the medical procedure. Each item is scored on a 4‐point Likert scale: (a) distraction, (b) pain, (c) request VR, (d) recommend VR, and (e) happiness with VR.

#### 2.4.7 HCP perception questionnaire

A 5‐item questionnaire modified from Ford et al. (2018) was used to assess HCPs' perception of the feasibility of the VR intervention by replacing words specific to burn wound care with the medical procedure. Each item is scored on a 4‐point Likert scale: (a) distraction, (b) pain, (c) interference with a medical procedure, (d) recommend VR, and (e) satisfaction with VR.

### 2.5 Observations, fieldnotes, and interview guides

#### 2.5.1 Observations and fieldnotes

The observation and fieldnote guide was developed by the research team and reviewed by HCPs prior to recording observations of the VR intervention ([Link], [Link], [Link] S1‐S3). Items notes included the following: (a) removal of VR headset; (b) interruptions to VR treatment; (c) use of pharmacological or nonpharmacological analgesia; (d) barriers and facilitators to VR implementation; (e) stakeholders' attitude toward VR; (f) interactions among HCPs and between HCPs and patients; (g) the impact of VR on clinical workflow; (h) pain and anxiety cues or request for pharmacological and nonpharmacological analgesics; (i) HCP and parental response to patients' pain and anxiety; and (j) reasons for discontinuing the VR intervention. As the VR headset covered part of the patient's face, only some of the pain and anxiety cues were observable.

#### 2.5.2 Semi‐structured interview guide

A brief, semi‐structured interview guide was developed by the research team and reviewed by HCPs prior to use ([Link], [Link], [Link] S1‐S3). The questions pertained to the feasibility, clinical utility (including acceptability and ease of use and understanding), and tolerability of the VR treatment from the child and parent perspective. Further, information was gathered on the patients' procedural pain and anxiety experienced during the VR intervention.

#### 2.5.3 Focus group guide

The guide was developed by the research team to collect data pertaining to the HCPs' perception of the VR intervention ([Link], [Link], [Link] S1‐S3). Questions and topics included: feasibility of using VR during medical procedures, HCP attitudes toward VR, advantages and disadvantages of VR, practicality issues, possible solutions to strategically implement the VR intervention in clinics, medical procedures for which VR was most beneficial (or not), who benefited the most from VR, and ways to improve VR clinical efficacy were discussed to better support HCPs.

### 2.6 Virtual reality intervention

**FIGURE 1:**
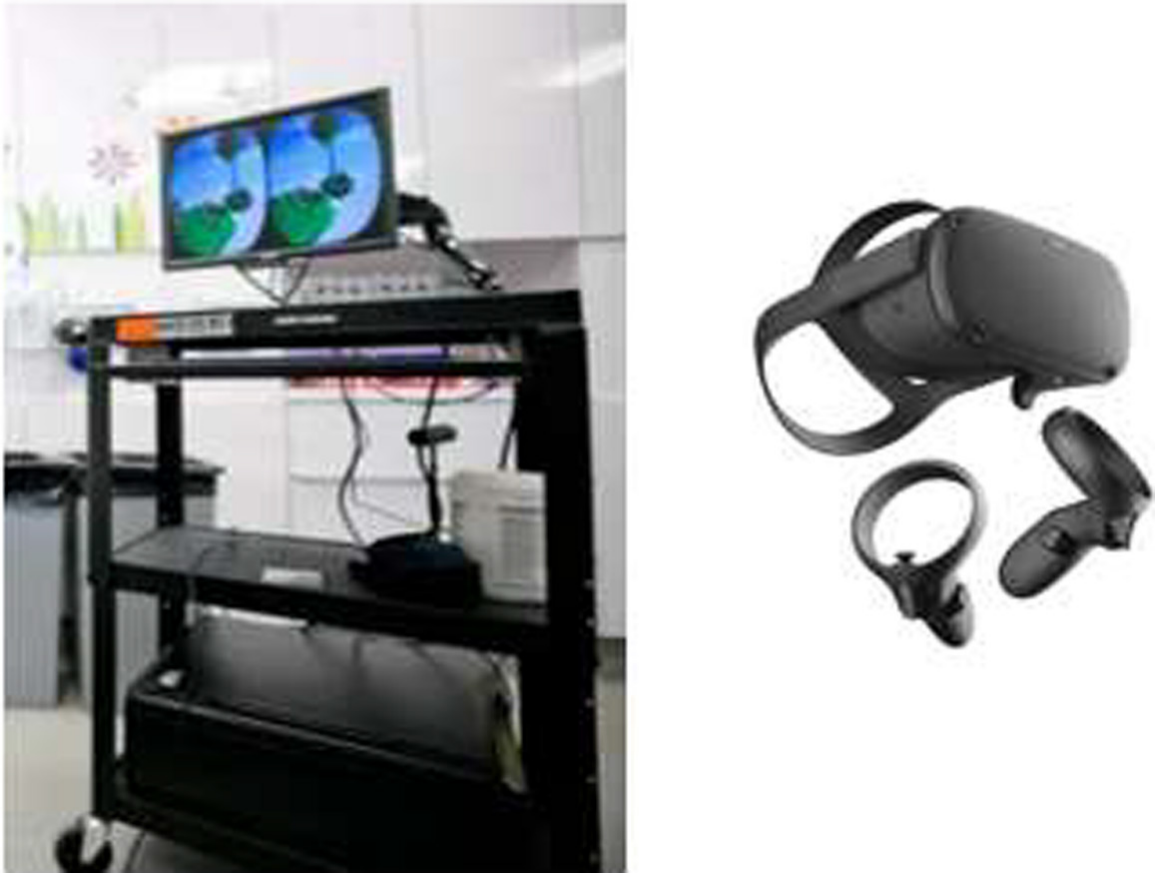
Virtual reality headsets used. *Note*: The Oculus Rift system is displayed on the left. This system consists of a computer monitor on a rolling cart with sensors, and a VR headset with a controller. The Oculus Quest is shown on the right. This system does not need to be powered by a computer, and only requires the use of a VR headset and a controller

Dreamland®, developed by Paperplane Therapeutics Inc., was the selected VR intervention. Dreamland® is a rigorously developed and tested VR game designed with and for children undergoing painful and anxiety‐inducing medical procedures (Paperplane Therapeutics Inc., 2016). The goal of the game is to throw red balls at balloons, diamonds, and purple trolls to accumulate points. Already, the game had been adapted to meet the needs of the healthcare setting by: (1) reducing the speed to prevent cybersickness, (2) requiring only one hand to play, and (3) using head movement only (i.e., no walking) to explore the virtual world. Patients could hear background music and special effects every time they scored a point. Throughout the study, two different VR systems were used, the Oculus Rift and the Oculus Quest (Figure 1). The Oculus Rift, used with the first six participants, consisted of a mobile cart with a monitor and sensors, along with the VR headset and controller. The Oculus Quest system was used with the remaining participants, which consisted of the VR headset and controller only. This change occurred as the Oculus Rift was deemed not feasible early on in the study as it occupied too much clinical space, took too much time to set up, and thus disrupted clinical workflow.

### 2.7 Data analysis

Descriptive statistics were used to analyze the sociodemographic and instrument data using the RStudio statistical software (Version 4.0.2 for Windows). Quantitative data analysis led to a list of key findings, which were set aside for comparison against qualitative findings at the interpretation phase of analysis. Directed content analysis was used to analyze qualitative data transcripts, allowing to identify and quantify repeating themes.^24^ Initially, a deductive approach was used to determine an initial list of codes derived from the VR‐CORE clinical outcomes. The code “acceptability” was grouped under the umbrella code “clinical utility,” defined as the acceptability, ease of use, ease of understanding, and satisfaction with VR. The codes “pain” and “anxiety” were listed under “initial clinical efficacy” as VR was used for pain and anxiety management. Operational definitions for each code were determined based on prior research and the VR‐CORE framework. Inductive coding was then used for data that did not fit into a predetermined code. Themes were then identified, with a focus on thematic frequency and patterns of similarities and differences between different stakeholder perspectives (patient, parent, and HCP). Quotes and observations were extracted from the data to support each theme.

Following the separate analysis of quantitative and qualitative data, the triangulation protocol ensued to integrate both components of the study.^25^ For each VR clinical outcome, major findings from quantitative and qualitative analyses were placed side‐by‐side on a table. This resulted in the creation of four convergence coding matrices, allowing for the comparison of quantitative and qualitative key findings. Each finding was given a triangulation code: convergence, divergence, complementarity, or silence.^25^ Convergence of findings from different data sources indicated strong validity. The divergence of findings invited further analysis to draw a conclusion. The complementarity of findings led to a more complete understanding. Findings generated only from one data collection method were coded as ‘silence.’ When possible, meta‐themes that cut across quantitative and qualitative data were identified while maintaining the three‐stakeholder perspective structure, and when necessary meta‐themes were broken down into sub‐themes. Integrated findings are presented together under themes or meta‐themes as supporting or conflicting evidence in the result and discussion sections, providing a rich and thorough interpretation of the findings.

## 3 RESULTS

### 3.1 Sample characteristics

**FIGURE 2:**
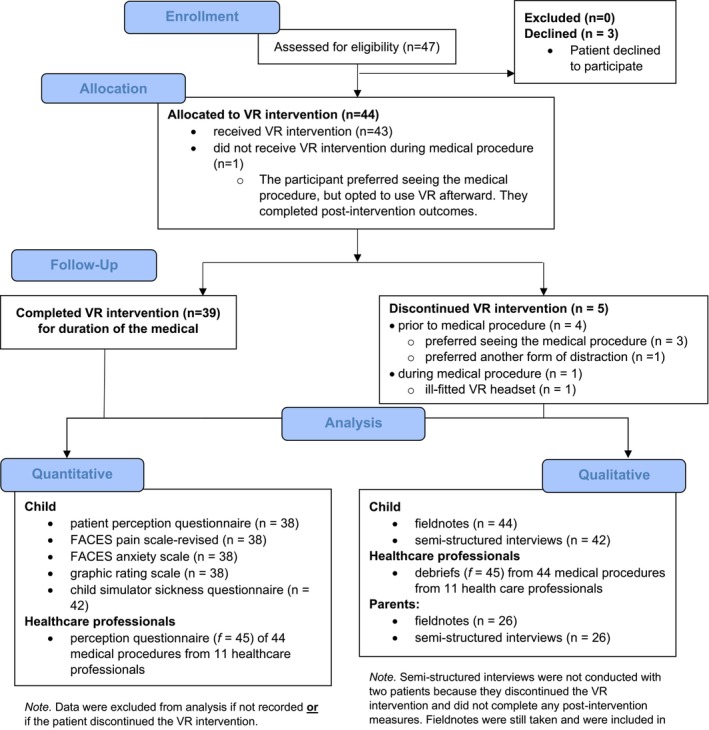
CONSORT flow diagram

In total, 47 eligible patients were approached for study participation and 44 patients consented and/or assented to participate in the study, for a participation rate of 94% (Figure 2). Twenty‐six patients' parents shared their perception of VR during the semi‐structured interviews. Patients' age ranged from 5 to 21 years old, with a mean age of 11.95 years old (SD = 4.18). There was an equal number of male (n = 22) and female (n = 22) participants (Table 1). The sample was diverse in medical diagnoses (n = 12) with the majority of participants diagnosed with osteogenesis imperfecta (OI; n = 30; 68.18%). Seven medical procedures were performed by either nurses or physicians, with at least one nurse present at all times and the majority of procedures were intravenous insertions (n = 30; 68.18%). Intervention adherence is summarized in Figure 2, and despite completion, discontinuation, or delayed use of the VR intervention, all study participants were included in the data analysis.

**TABLE 1:** Sample characteristics

N = 44	
Age (years)	
Mean	12.0
Standard deviation	4.2
Range	5‐21
Sex (%)	
Male	22 (50.0)
Female	22 (50.0)
Race/ethnicity (%)	
African American/Black	3 (6.8)
Asian	1 (2.3)
Caucasian	37 (84.1)
Hispanic/Latino	1 (2.3)
Unknown	2 (4.6)
Medical diagnosis (%)	
Bilateral club feet	1 (2.3)
Cerebral palsy	1 (2.3)
Charcot Marie tooth	4 (9.1)
Duchenne’s muscular dystrophy	1 (2.3)
Joint stiffness of the knee	1 (2.3)
Leg length discrepancy	1 (2.3)
Legg‐Calve‐Perthes disease	1 (2.3)
Osteogenesis imperfecta	30 (68.2)
Osteoporosis	1 (2.3)
Neurofibromatosis	1 (2.3)
Spina bifida	1 (2.3)
Syndrome Aicardi‐Goutieres	1 (2.3)
Medical procedure (%)	
Blood draw	3 (6.8)
Botox injections	2 (4.6)
Dressing change	1 (2.3)
Intravenous injection	30 (68.2)
Pin removal	7 (15.9)
Urodynamics	1 (2.3)

Eleven HCPs performed or assisted with a medical procedure during the VR intervention. The majority of these medical procedures (n = 27) were performed by one nurse (HCP‐01) in the Medical Day Center. However, ten other HCPs conducted or assisted other medical procedures during VR distraction in a variety of clinics across the study site (Table 2).

**TABLE 2:** Healthcare professionals characteristics

HCPs	Position	Practice location	# Procedures completed	Types of procedures completed
HCP‐01	Nurse	Medical day center	27	IV treatment Blood draw
HCP‐02	Nurse	Cast room Medical day center	8	Pin removal IV treatment
HCP‐03	Child life specialist	Medical day center Outpatient clinic	3	IV treatment Botox injections
HCP‐04	Nurse	Cast room Inpatient clinic	2	Pin removal Dressing change
HCP‐05	Nurse	Inpatient clinic	1	Blood draw
HCP‐06	Nurse	Cast room	2	Pin removal
HCP‐07	Nurse	Medical day center	3	IV treatment
HCP‐08	Nurse	Outpatient clinic	1	Botox injection
HCP‐09	Doctor	Outpatient clinic	1	Botox injection
HCP‐10	Nurse	Outpatient clinic	1	Urodynamic test
HCP‐11	Nurse	Inpatient clinic	1	IV treatment

*Note*: Some procedures required more than one HCP.

Abbreviation: HCP, healthcare professional.

### 3.2 The feasibility, clinical utility, tolerability, and initial clinical efficacy of virtual reality use during medical procedures

Guided by the VR‐CORE Model framework,^17^ the following VR clinical outcomes were identified: feasibility, clinical utility, tolerability, and initial clinical efficacy. Each outcome was further analyzed and organized into themes or meta‐themes.

### 3.3 Feasibility

Fieldnotes of stakeholder observations, HCP debriefs, patient interviews, and completed HCP Perception Questionnaires were integrated into two meta‐themes and one theme.

#### 3.3.1 The VR intervention was compatible with the completion of different medical procedures

Most of the time (f = 41 procedures, 91%), HCPs reported that VR did not interfere at all with their ability to complete the medical procedure (Table 3). This was corroborated in observations of VR use, where HCPs completed medical care as usual. In some rare cases, HCPs adapted their standard protocol to accommodate the restrictive position imposed by the VR intervention. For instance, a physician administering Botox injections would have preferred the patient to be lying down for the procedure. The physician, however, adapted the procedure, indicating the VR intervention only interfered “a little bit” with their ability to complete patient care. Further, during a urodynamic test, where the child had to lie down to maintain proper bladder pressure, a pillow was placed behind the participant's head to improve their view of the VR world. Finally, a child receiving a zoledronate IV treatment was asked by the nurse to keep their arm still while playing the VR game, as they were deeply engaged in the VR world. Some patients (n = 5) shared during interviews the headset was too big or heavy. This led to adjustments or removal of the headset altogether before resuming the medical procedure. Despite causing some observed interruptions to care, no HCPs reported that an ill‐fitted VR headset interfered with their ability to complete a medical procedure.

**TABLE 3:** Healthcare professional perceptions of the VR intervention feasibility (*f* = 45)

Healthcare professional perception questionnaire items	Mean (SD)
1. From your perspective, how much did the virtual reality experience distract the patient during the medical procedure?	2.5 (0.9)
2. From your perspective, how much did the virtual reality experience help decrease your patient’s pain and distress during the procedure?	2.4 (1.0)
3. Did the patient’s use of virtual reality interfere with your ability to complete your patient’s care?	0.2 (0.7)
4. Overall, if it were an option, would you recommend using virtual reality for this particular patient for future care?	2.6 (0.8)
5. Overall, how satisfied were you in using virtual reality for this particular patient?	2.6 (0.8)

*Note*: A total of 45 questionnaires were filled out by healthcare professionals (n = 11). Each item is scored from 0 to 3. For items 1‐3: 0 = not at all; 1 = a little bit; 2 = some; 3 = a lot. For item 4: 0 = not at all; 1 = possibly; 2 = probably; 3 = definitely. For item 5: 0 = very unsatisfied; 1 = unsatisfied; 2 = satisfied; 3 = very satisfied.

#### 3.3.2 The VR intervention was well‐integrated into the clinical workflow

The total length of the VR intervention, from set up to clean up, was on average 12.7 minutes (SD = 7.2 minutes). However, when comparing the two VR systems used, the Oculus Quest was much faster to set up (mean = 1.3 minutes) compared to the Oculus Rift (mean = 6.5 minutes), and thus was more seamlessly integrated into clinical workflow. The shift was evident as nurses used to have to wait, sometimes frustrated, for the Oculus Rift to start‐up, while the Quest headset was ready for immediate use. The time difference was especially noted during back‐to‐back use of the Oculus Rift VR with different patients, further slowing down the clinical workflow, and also limiting the availability of VR to all patients receiving care at the study site.

In a busy clinic, with a 1:4 HCP to patient ratio, HCPs were observed prioritizing the completion of medical procedures efficiently over the quality of the VR intervention. One HCP shared, “the only thing I am concerned with is, in the future, it *[VR intervention]* has to be very simple” or else nurses will be reluctant to use VR during busy clinics. This led to insufficient preprocedure VR playtime for effective immersion and distraction. The consequences were highlighted during a pin removal procedure, during which the child's verbal and physical display of pain reminded the nurses that giving sufficient VR playtime for immersion before the start of the procedure was necessary to achieve effective and immersive distraction. Similarly, another child explained that with more preprocedure VR playtime, they would have better understood the game and likely not removed the VR headset during the medical procedure.

#### 3.3.3 The VR intervention allowed for communication between stakeholders

Communication was observed to be an integral part of nurses' and physicians' current workflow, as they often checked in with their patients throughout the medical procedure while the patient was immersed in VR, explained what they were doing, or gave instructions. While some patients were curious to know what was happening while they were immersed in VR, others insisted their nurse or physician warn them before starting the medical procedure. One patient asked, “Can I count down from three. I don't like surprises” (Child Participant‐13) before the nurse inserted their IV. By being able to communicate with their HCP, patients seemed more at ease and in control. Moreover, HCPs were actively involved in the VR intervention, asking patients what they were seeing or encouraging them, “Are you shooting all the purple monsters?” (HCP Participant‐06). This fostered a positive and safe environment for patients to be immersed in VR, Likewise, it was important for patients to share their excitement with their parents, HCPs, and the researcher. HCPs could still communicate with each other to complete patient care together, as seen during medical procedures requiring more than one HCP, such as IV insertions, Botox injections, and pin removals.

### 3.4 Clinical utility

Clinical utility was evaluated through observations, questionnaires, semi‐structured interviews, and focus groups, generating the following themes and meta‐themes.

#### 3.4.1 Stakeholders were willing to use VR during a medical procedure

All patients and their accompanying parents were accepting of VR use as shown through their willingness to try the VR intervention during their care, regardless of their familiarity with VR. Patients were seen exploring the virtual world by moving their heads around and using the controllers to score points. This aligned with patients' likeliness (n = 37; 97.4%) to request VR at following appointments, incorporating the innovation into their usual care (Table 4). Similarly, parents witnessed the value of VR in distracting their child, requesting it throughout their care, “I would prefer to, you know, have this game next time as well. Cause I, I felt like he did much better” (Parent Participant‐23). HCPs were accepting of VR and saw VR as a way of improving care, seen through their active participation and engagement in the intervention, asking patients to describe what they saw, and encouraging them to score points. Nurses reserved VR use ahead of time and the child life specialist integrated VR into their arsenal of distraction tools for patients.

**TABLE 4:** Patient perceptions of the clinical utility of the VR intervention (N = 38)

Patient perception questionnaire items	Mean (SD)
1. How much did the virtual reality game distract you during your medical procedure?	2.5 (0.7)
2. How much did the virtual reality game help lower your pain during your medical procedure?	1.7 (1.0)
3. Would you ask to play a virtual reality game for your next medical procedure?	2.6 (0.6)
4. Would you recommend playing a virtual reality game to another patient like you?	2.8 (0.4)
5. How happy were you with playing the virtual reality game during your medical procedure?	2.7 (0.5)

*Note*: Each item is scored from 0 to 3. For items 1‐2: 0 = not at all; 1 = a little bit; 2 = some; 3 = a lot. For items 3‐4: 0 = very unlikely; 1 = unlikely; 2 = likely; 3 = very likely. For item 5: 0 = very unhappy; 1 = unhappy; 2 = happy; 3 = very happy.

In six cases, acceptability for VR was withdrawn by the patient as VR was only increasing their procedural anxiety. For instance, one participant, despite being familiar with VR, tensed their body and moved closer to their mother during the intervention, showcasing some patients may, “… prefer seeing [the medical procedure] … [because they] just feel more comfortable” (Child Participant‐39). Similarly, four participants (9.09%) began the VR intervention, but then asked to remove the VR headset to watch the procedure, as a way of coping with their anxiety, “it *[watching the medical procedure]* kinda helps my stress so I'm like not ‘oh no when is it *[IV insertion]* coming, when is it coming’” (Child Participant‐11) One patient, rating VR as “a little bit” helpful in distracting them and reducing their pain, abandoned VR, choosing to play a videogame they brought to their appointment.

#### 3.4.2 The VR intervention was easy to use and understand

Regardless of age and previous VR experience, most patients described their VR experience as ‘easy’ and “simple” reporting no trouble with understanding how to work the game. As one participant shared, the ease of use with the VR intervention was likely due to its resemblance to other typical video games, “…it was really easy. It looks a lot like other videogame controllers, so… I quickly got the gist of it” (Child Participant‐38). In rare cases, patients thought VR was too complicated and it was hard to aim or shoot objects in the game, or the game was moving too fast, but only led to one cessation of VR. Administering the VR intervention was straightforward; however, the Oculus Quest VR system was easier to use in a clinical context because it used minimal space and time to set up.

#### 3.4.3 Stakeholders were satisfied with and saw the benefits of VR

Patients were seen enjoying the VR game, with most being “happy” or “very happy” with their VR experience (n = 37, 84.10%) (Table 4). Satisfaction was related to patients' perceived usefulness of VR as a distraction from anxiety, with reports of VR's distraction ability as “somewhat” or “a lot” (n = 33; 86.84%) (Table 4) and the feeling of being immersed and engaged in the virtual world. However, a lack of engagement in the game, and thus dissatisfaction, was encountered whether VR was considered not age‐appropriate or too easy.

Overall, parents were satisfied with VR, with some even expressing gratitude, “thankfully we had VR, or else it *[the medical procedure]* would have been a lot harder” (Parent Participant‐06). This highlights that procedural pain or anxiety is a concern for parents, and VR is perceived as a useful intervention. Dissatisfaction from parents, albeit uncommon, was associated with perceived insufficient preprocedural playtime, “I think that giving [my child] more time to play before … [they] would have had more time to get into the game. And maybe [they] wouldn't had ‐ well, I am not saying that she wouldn't of had noticed it *[medical procedure]* at all ‐ but maybe less” (Parent Participant‐24).

#### 3.4.4 Stakeholders would recommend VR during medical procedures

The majority of patients (n = 37; 84.1%) were “likely” or “very likely” to recommend the VR intervention to another patient (Table 4). Reasons for recommending VR included the perceived distraction and stress‐relieving benefits of VR. Parents reported that VR could be helpful for their other children and even during other orthopedic procedures. Finally, HCPs would also recommend VR distraction to their patients again (f = 84.44%) (Table 3), but were less likely to do so with very anxious or older patients (f = 15.55%), and some suggested more age‐appropriate games (Table 3).

### 3.5 Tolerability

Tolerability was assessed using semi‐structured interviews and questionnaire data. The following themes were synthesized.

#### 3.5.1 Absence of cybersickness

Before starting VR, most participants showed no visible signs of sickness and the majority (n = 39; 92.9%) reported no sickness as measured by the Child Simulator Sickness Questionnaire (CSSQ) (Table 5). When the CSSQ indicated simulator sickness for a participant (n = 3), they were given the option to proceed with the intervention, and they all did with no further tolerance issues. While no patient scored for simulator sickness on the CSSQ during VR, a patient reported, “it was as if I was on a ride, and I felt a bit nauseous” (Child Participant‐06) and another explained, “I felt a bit dizzy cause I was like floating in the sky” (Child Participant‐12). Two children complained of blurry vision while playing, but adjustment of the headset alleviated this symptom.

**TABLE 5:** Comparison of child simulator sickness scores before and after the VR intervention (n = 42)

Score	Pre‐VR n (%)	Post‐VR n (%)
<3	39 (92.3)	42 (100.0)
≥3	3 (7.1)	0 (0.0)

*Note*: A score of ≥3 of the CSSQ indicates the presence of simulator sickness.

#### 3.5.2 Absence of emotional adversity

The vast majority of patients enjoyed using VR during their medical procedure, as shown with an average “fun” rating of 8.4/10. Some were familiar with VR, while others were eager to try VR for the first time. Technology anxiety was visible in a minority of patients (n = 3), but whether present, the anxiety led to VR refusal or interruptions, as seen with one child who removed the VR headset because she preferred watching her medical procedure.

#### 3.5.3 VR equipment may cause discomfort

Six children (aged 5‐8 years) with osteogenesis imperfecta said the VR headset was too big or too heavy. Patients with this diagnosis typically have a smaller stature overall, making it challenging to achieve optimal fit of the headset on their head, ultimately causing discomfort. This led to the removal of the VR headset and the complete cessation of the intervention for two participants. Others proceeded with the intervention but complained that “It *[VR headset]* was falling down… I had to keep it up myself” (Child Participant‐24). Reasons for removal of VR headset included personal preference, needing to blow nose, itchy eyes, and removal of sweaters for the procedure.

### 3.6 Initial clinical efficacy

The main patient‐reported outcomes assessed to describe the initial clinical efficacy of VR were procedural pain and anxiety. However, distraction was added as an outcome through inductive analysis. The follow themes and meta‐themes were generated.

#### 3.6.1 Patients experienced some pain during the VR intervention

**FIGURE 3:**
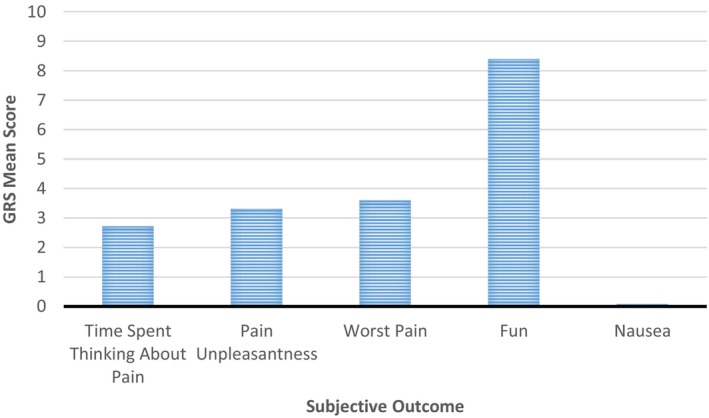
Patient’s self‐reported procedural pain using a graphic rating scale (N = 38)

The average pain self‐reported by patients using the FPS‐R at baseline was 0.8 (SD = 1.6; R = 0‐6), and increased to an average of 3.5 during the medical procedure (SD = 3.3; R = 0‐10) (Table 6). As most patients (n = 29; 76.3%) reported no pain at all at baseline, this increase in pain highlights the acute nature of the pain as it stemmed from the procedure itself. Further, patients rated their worst procedural pain as “mild‐to‐moderate” (mean = 3.6; SD = 3.4; R = 0‐10) and as “mildly unpleasant” (mean = 3.3; SD = 3.5; R = 0‐10) (GRS; Figure 3). Participants spent “some of the time” thinking about their pain during the medical procedure and VR intervention (mean = 2.7; SD = 2.5; R = 0‐10) (Figure 3). These signs of covert pain were consistent with overt pain behaviors patients displayed during the medical procedure and VR intervention, with 61.4% (n = 27) of patients exhibiting at least one overt pain cue. Most often, patients verbally expressed their pain (n = 14; 31.8%) by saying “it hurts,” or “ouch!” Nonverbal pain behaviors were less common but included: uneasiness or tenseness (n = 12; 27.3%), facial expression (n = 11; 25.0%), withdrawing (n = 8; 18.2%), and crying or distress (n = 8; 18.18%). Moreover, some patients (n = 6) momentarily stopped engaging in the VR game during the medical procedure due to their pain, signaled by a pause in head movement and letting go of the controller. During these painful moments, parents comforted their children by patting them on the back, telling them the medical procedure was almost over, or encouraging them to keep playing the VR game. Overall, however, most patients (52.5%) reported minimal levels of pain (FPS‐R score = 0‐2) while engaging in VR.

**TABLE 6:** Patients' self‐reported pain and anxiety before and during the medical procedure (N = 38)

	Mean ± Standard deviation (Range)
Baseline pain	0.8 ± 1.6 (0‐4)
Procedural pain	3.5 ± 3.3 (0‐10)
Anticipatory anxiety	1.1 ± 1.1 (0‐4)
Procedural anxiety	0.8 ± 1.0 (0‐4)

*Note*: Pain was measured using the FACES Pain Scale–Revised.

Anxiety was measured using the FACES Anxiety Scale.

#### 3.6.2 Stakeholders lacked consensus about the ability of VR to manage procedural pain

The analgesic benefit of VR during medical procedures was only voluntarily shared by four patients, with one child saying that “[VR] took away the pain” (Child Participant‐24). Other patients (n = 6) specified that distraction provided by VR was the main reason for pain relief, “the fact that I have something else to look at is really helpful” (Child‐Participant‐18). One child completely disagreed, saying, “No. I still felt everything” (Child Participant‐23). These mixed reports were further supported by patients' perception of the VR game in lowering their procedural pain, with answers distributed between “a lot” and “not at all” (Table 4). In contrast, most parents and HCPs thought VR helped reduce their child or patient's pain. For parents, they were able to compare past procedures done without VR, “[they were] yelling for a lot less time… I saw that things got better much quicker. That's right because the last time [they] got her pins removed, [they were] in pain for a long time!” (Parent Participant‐24). Only one parent disagreed, saying they preferred pharmacological interventions during their child's urodynamic tests.

#### 3.6.3 Patients experienced low anticipatory and procedural anxiety and used various coping mechanisms

Most patients (n = 30; 68.2%) self‐reported anticipatory anxiety at baseline, with an overall average of 1.1 (SD = 1.1; R = 0‐4) using the FAS at baseline (Table 6). Albeit relatively low, anticipatory anxiety was observed through the anxiety behaviors patients exhibited prior the start of the medical procedure, including uneasiness or tenseness (n = 12; 27.3%), facial expression (n = 11; 25.0%), withdrawing (n = 8; 18.2%), crying or distress (n = 8; 18.2%), and restlessness or agitation (n = 4; 9.1%).

As for procedural anxiety during the VR intervention, patients self‐reported an average of 0.8 using the FAS (SD = 1.0; R = 0‐4). This relatively low average was supported by verbal reports, “well it [VR] helps reduce stress” (Child Participant‐02) and “It [VR] takes away a lot of the stress” (Child Participant‐38). Anxiety‐related behaviors were still present with VR for some patients. For instance, some patients repeatedly inquired about the medical procedure during the VR intervention (n = 2), insisted the HCP warn them before starting the medical procedure (n = 3), removed the VR headset as soon as they felt the HCP (n = 2), or altogether stopped the VR intervention to watch the medical procedure (n = 4). Some patients used their own tried and true coping mechanisms, anxious by the novelty of VR, including deep breathing, parental comfort, and control. One child shared, “It's not that I don't like VR. It's just that I like seeing what is going on” (Child Participant‐11). That being said, about half of patients (n = 19; 47.5%) reported no procedural anxiety at all.

#### 3.6.4 Parents and HCPs thought VR helped manage anxiety

For patients who had previous anxiety‐inducing experiences at the hospital, parents noticed their child was more relaxed and less stressed with VR, “yeah, [they] would normally be like that [pointing to their other child crying]” and “I didn't think [they] would be this calm” (Parent Participant‐28). Parents further shared that having VR to focus on instead of the medical procedure helped reduce their child's anxiety, “well I think it [VR] helped a lot because usually [they] has a lot of apprehension towards medical procedures … [they have] been thinking about it for 2‐3 days. Even earlier, [they] didn't want to do it [pin removal procedure], so thankfully now we have VR or else it would have been harder” (Parent Participant‐06). Anxiety relief from VR was continued after the end of the medical procedure, with some parents describing their child as more calm because they could focus their attention on the game, rather than the medical procedure, “I would say that right after [the pin removal procedure they were] much calmer, and she got back into the game right away” (Parent Participant‐24).

#### 3.6.5 VR was immersive and provided a great distraction

Across stakeholders, distraction was the most beneficial quality of VR. The majority of patients (82.5%) reported that VR helped distract them from the medical procedure ‘a lot’ or ‘some’ (n = 10; 25.0%) (Table 4). This was further conveyed during participant interviews, “It was like in the back of my mind that I was having it [the procedure] but in the front of my mind I was playing the game” (Child Participant‐12). Patients recognized that VR helped shift their focus, making them forget about the procedure, “I barely paid attention [to the IV insertion] because I was so focused on the game … it [VR] really diverts your attention” (Child Participant‐38). Distraction, for some, was powerful because they felt a sense of presence in the VR game, “well I think that overall, it [VR] is immersive” (Child Participant‐38).

Parents concurred, “[They were] less anxious and more distracted with the game instead of focusing on what's going on with [their] wrist. I think he did much better” (Parent Participant‐23). Parents also noticed that their child was engaged and immersed in the VR world, “and then [they were] still looking around, playing the game. [They were] more distracted” (Parent Participant‐35). Other parents agreed, “It was a very good distraction … the fact that [they are] completely immersed in it…” (Parent Participant‐30) and “You could tell [they were] engaged in the game” (Parent Participant‐35). During debriefs with HCPs, they also were impressed with the immersive quality of VR. However, HCPs also highlighted that VR distraction may not be efficacious whether the patient had a past trauma related to the medical procedure. The Child Life Specialist explained for one particular patient, “The patient was extremely anxious upon arrival. [They] was crying. The patient had experienced a past traumatic experience where [they] received ten pokes. Therefore, [the patient] still remembers [their] past experience and it was difficult for [them] to focus on the virtual reality” (HCP Participant‐03).

## 4 DISCUSSION

This study examined the feasibility, clinical utility, tolerability, and initial clinical efficacy of VR during painful or anxiety‐inducing medical procedures received by children and adolescents with complex musculoskeletal conditions. The results reflect a triad perspective from key stakeholders – patient, parent, and HCPs – showcasing the feasibility of VR depends on its compatibility with medical procedures, clinical setting, and communication. VR was clinically useful as depicted by stakeholders openly welcoming and requesting VR during care. The VR intervention did not cause serious adverse effects, but an ill‐fitted VR headset reduced tolerability. Most patients reported low levels of pain and anxiety both at baseline and during their medical procedure. However, verbal and behavioral expressions of pain and anxiety were exhibited by some patients throughout the medical procedures. Finally, stakeholders agreed that distraction was the most beneficial aspect of VR, suggesting a new outcome of the clinical efficacy of VR.

Our results, showcasing VR feasibility at the study site, are consistent with the literature.^16^, ^22^, ^26^, ^27^, ^28^, ^29^, ^30^, ^31^, ^32^, ^33^, ^34^ Contrary to our results, however, some studies reported that VR consumes significant clinical time, with some needing 10 minutes to set up and clean up the VR equipment.^31^, ^32^ The use of a stand‐alone VR system only requiring a VR headset, such as the Oculus Quest used in the present study, saved time and thus more easily implemented within the clinical workflow.^22^, ^28^ Similar to Ford et al. (2018), VR did not interfere with the completion or delivery of usual care showcasing the feasibility of introducing VR into practice through excellent partnerships with clinicians. Agrawal et al. (2019) reported child life specialists played an important role in facilitating and encouraging the use of VR, which was found in the present study as well. While to our knowledge no other study has described the state of the clinical environment during VR use, a busy clinic may lead to a low‐quality intervention due to insufficient immersive play and reduced procedural compliance. Finally, consistent with other studies, VR maintained a usual patient and HCP communication, allowing patients to set boundaries regarding interventions, such as being warned before the start of the procedure, stopping the intervention, sharing their excitement, or requesting the use of other coping mechanisms.^33^, ^34^

Consistent with other studies, VR was clinically useful during painful and anxiety‐inducing medical procedures. All stakeholders deemed VR was acceptable to use during medical care, as seen with their willingness to try and use VR again at their next appointment, as reported elsewhere.^22^, ^29^, ^32^, ^33^, ^34^, ^35^ Similar to other studies, lower acceptability due to high patient anxiety or technology anxiety was observed.^22^, ^35^ However, only a minority of our participants (n = 6; 13.64%) refused or discontinued the VR intervention during their medical procedure. As reported in Ford et al. (2018), when VR did not coincide with patient preferences for watching their medical procedure, patients were less likely to consider VR as a coping tool. Mosadeghi et al. (2016) reported on the digital divide, with younger individuals more willing to use VR; however, we observed that age was not a factor in VR acceptability, with patients up to 21 years old willing to participate. Moreover, consistent with other studies, patients and HCPs were satisfied or happy with VR and would recommend VR to other patients.^22^, ^26^, ^33^, ^34^, ^35^ Patients especially enjoyed VR because it was an immersive, interactive, and distracting experience.^22^, ^26^, ^33^, ^34^, ^35^ This implies that immersive and interactive VR experiences may provide the optimal environment for patients to forget about their medical procedure. An ill‐fitted VR headset is reported to reduce the ease of use as it may fall down during the procedure.^33^

Minimal physical or emotional adverse events were reported by patients during the VR intervention. This may be the result of using Dreamland®, a VR game that was specifically designed with reduced speed, thereby decreasing the risk of cybersickness documented in other studies.^30^ Simulator sickness is inconsistently reported in the literature with some researchers reporting simulator sickness and others finding no issues.^32^, ^33^, ^35^ Consistent with other studies, common tolerability complaints are ill‐fitted and heavy VR headsets.^33^, ^35^ These complaints were especially marked in our osteogenesis imperfecta sub‐sample, which characteristically has a smaller stature. This may cause discomfort to patients and reduce the quality of the VR intervention as the VR headset may fall down during the procedure.^33^

Present descriptive study findings showcase that the mean self‐reported procedural pain was relatively low during the VR intervention; however, the reported mean was higher than that measured in other needle‐related pain studies.^30^, ^36^, ^37^, ^38^, ^39^, ^40^, ^41^, ^42^, ^43^, ^44^, ^45^, ^46^, ^47^, ^48^, ^49^ This observation may be explained by the medical procedures included in the study, such as pin removals (n = 7) and Botox injections (n = 2), and IV insertions with a population known to have challenges with venipuncture (n = 30), which have not been previously tested for VR efficacy. Such procedures may be perceived as more painful and thus may be more feared, leading to increased anticipatory anxiety, which in turn may increase the painful experience. Few studies assessed the multidimensional aspects of pain during VR. Atzori et al. (2018) reported lower sensory (mean = 2.00, SD = 1.20), affective (mean = 0.93, SD = 1.16), and cognitive (mean = 1.33; SD = 1.05) pain during venipuncture in children.^49^ Two studies assessed affective pain during a needle‐related procedure, defined as the worry and bother related to pain, and reported lower affective pain levels.^30^, ^43^

The present descriptive study findings demonstrate relatively low levels of anticipatory and procedural anxiety with the VR intervention. Studies measuring pain‐related fear during a medical procedure found low scores.^39^, ^40^, ^41^, ^47^ However, contrary to the current study, the scores for anxiety prior to the medical procedure were moderate. Similarly, other studies evaluating anticipatory anxiety reported moderate scores, using various scales.^40^, ^50^, ^51^ As for procedural anxiety levels, other studies also report similar low scores.^27^, ^30^, ^37^, ^38^, ^41^, ^44^, ^51^ Piskorz and Czub (2018) and Piskorz et al. (2020) specifically measured stress intensity during the medical procedure and found low stress levels with a VR intervention, which was corroborated in the present study during patient interviews.

The literature shows that children's previous experience with pain influences their current expectation of pain during a medical procedure.^52^ This was observed in children with past traumatic experiences with a painful medical procedure who were fearful and anxious about the upcoming painful event and rated their perceived procedural pain high. Children may also rely on their parents or HCP to help them get through a painful or anxiety‐inducing experience,^52^, ^53^ as observed with children seeking emotional or physical comfort from their parents. Children need to feel secure and have a sense of control before being effectively distracted.^52^, ^53^ For example, children were observed setting boundaries with their nurse for their medical procedure and VR intervention, such as requesting warning signals. Once a sense of trust was established, children were more at ease and able to engage with the VR distraction. Similarly, when investigating the efficacy of VR during burn wound care with children, Ford et al. (2018) found that children want to voluntarily use VR and need to feel secure to stop the intervention at any time by openly communicating with healthcare professionals. In the present study, when children felt a lack of control over their medical procedure, some momentarily removed their VR headset to see their procedure, while others permanently removed their VR headset for the remainder of their procedure to attentively observe the procedure. Instead of distracting them, VR increased their anticipatory anxiety. This coping behavior was also seen in other studies using VR during needle‐related procedures with children.^28^, ^41^, ^42^, ^51^

### 4.1 Clinical implications

The use of VR in healthcare settings should be guided by policies and procedures grounded in research findings. As child life specialists already offer distraction and coping tools to patients, they would be ideal champions for VR use. The clinical environment should be scanned for the feasibility of the VR intervention, considering space and time resources depending on the VR system used. VR should be presented as one possible coping tool for procedural pain and anxiety. If a patient has a known history of anxiety or past traumatic experiences relating to medical procedures, the HCP should assess whether VR would benefit the patient or cause more anxiety. Healthcare professionals should be sensitive to pain and anxiety cues specific to VR, including reduced activity and movement of the head and hands. Further, the time point at which the patient would like to use VR should be discussed, as some patients may only benefit from using VR before, during, and/or after their procedure. A communication plan should be outlined, considering whether the patient prefers step‐by‐step explanations of the medical procedure, warnings before inserting a needle, check‐ins, or complete immersion in the VR world. Finally, proper fit of the VR headset is essential for a smooth and tolerable intervention with no interruptions to the completion of care.

### 4.2 Recommendations for future research

For a hospital‐wide implementation of VR, future studies should continue to examine the feasibility, clinical utility, tolerability, and initial clinical efficacy of the VR intervention considering their own organizational context. This would allow for the identification of important considerations and accommodations needed for medical procedures that require further testing such as Botox injections and pin removals.^54^ Next studies should transfer the responsibility of administering the VR intervention to HCPs to replicate real clinical conditions. History of anxiety and trauma‐related to medical procedures should be collected to investigate the feasibility and clinical utility of VR with these patients. Future studies should opt to use VR systems that are mobile and ready for immediate use, to best suit clinical needs and reduce potential interruptions. Considering VR pain and anxiety relief is mediated by the distraction quality of VR, the use of validated scales to measure distraction during VR is warranted. This would help further the understanding of the efficacy of active VR distraction compared to passive distraction and generalize study findings. Finally, physiological indicators of pain and anxiety could strengthen self‐reported and observed data; however, more studies should collect qualitative data to describe children's procedural pain and anxiety experiences as these remain contextual and subjective matters.

### 4.3 Strengths and limitations

The mixed‐methods study allowed for the comprehensive description of the use of VR at the study site and the integration of quantitative and qualitative findings through triangulation. This produced an enriched understanding of the research question, something that many studies fail to do.^18^ Further, for the first time, VR was tested for feasibility and initial clinical efficacy during pin removals, Botox injections, and urodynamic tests, which are procedures commonly done at the study site. Finally, this study shares the perspectives of three stakeholders, gaining a holistic understanding of VR use in healthcare. The study was conducted at a tertiary pediatric hospital specialized in musculoskeletal care, liming the generalizability of the results. The majority of study participants had an osteogenesis imperfecta diagnosis and underwent a needle‐related procedure. While acceptability ratings were high among HCPs, most medical procedures were completed by one nurse. This may skew the findings as with time and practice, HCPs may become more receptive to VR and have better integrated the technology in their clinic. Moreover, the VR software used, Dreamland, is designed for children; however, our sample included patients up to the age of 21 years. The VR headset occupied part of the children's face, potentially covering some pain and anxiety cues. Further, the data collected do not allow for conclusions to be made regarding the clinical efficacy of VR. Rather, as outlined by the VR‐CORE Model framework, initial clinical efficacy measures were meant to gather initial insights for a future, rigorous randomized controlled trial.

## 5 CONCLUSION

Finally, the study showcased that the use of VR distraction is feasible, clinically useful, and tolerable to key stakeholders (patients, parents, and HCPs) during painful and anxiety‐inducing procedures. This early success is in part explained by the compatibility of VR with the medical procedures performed at the study site, an openness from stakeholders to try and adopt new technologies, and the use of carefully designed VR software to reduce the risk of adverse effects. Results contribute to the growing body of evidence for the benefits of VR distraction with children in the hospital setting, adding new descriptive data on VR use during pin removal, Botox injections, and urodynamic tests. Policies and procedures for VR use will be delineated from study findings to ensure a sustainable implementation across [the name of hospital removed for peer review] network.

## Supporting information


**Appendix** S1

**Appendix** S2

**Appendix** S3
