# Supplementary material for: The use of virtual reality during medical procedures in a pediatric orthopedic setting: A mixed‐methods pilot feasibility study
Source: Paediatr Neonatal Pain. 2022 Apr 14;6(3):45–59. doi: 10.1002/pne2.12078 (PMC11514299; doi:10.1002/pne2.12078)
Supplement: Supplementary file 1 — Appendix S1 [file PNE2-6-45-s003.docx]

**Appendix S1**

**Fieldnotes and Observations**

**Time point 1 (VR intervention/medical procedure – T1)**

Time the VR intervention from the start of the equipment set up (round to the nearest minute) – completed by RA

1. Set up: _____ mins
2. VR intervention: _____ mins
3. Clean up: _____ mins

(4) Did the child keep the HMD on the whole time? YES 🞎 NO 🞎

- If no, specify: ____________________________________________________________________________________________________________________________________________

(5) Was the VR intervention interrupted? YES 🞎 NO 🞎

- If yes, specify: ____________________________________________________________________________________________________________________________________________

(6) Was there a need to use another non-pharmacological intervention? YES 🞎 NO 🞎

- If yes, specify: ____________________________________________________________________________________________________________________________________________

(7) Was there a need to use a pharmacological intervention? YES 🞎 NO 🞎

- If yes, specify: ____________________________________________________________________________________________________________________________________________

(8) Were there any of the following verbal or non-verbal cues of pain or anxiety during the VR intervention/medical procedure? YES 🞎 NO 🞎

🞎 facial expressions (grimace, frown, etc.) 🞎 crying or distress

🞎 withdrawing 🞎 restlessness or agitation

🞎 guarding area in pain 🞎 uneasy or tense

🞎 other: ________________________________________________________________________________________________________________________________________________________

**FEASIBILITY: Implementation Barriers and Facilitators**

| **Barriers/Facilitators** | **Cause** | **Solutions** |
| --- | --- | --- |
| **Patient** |  |  |
| **Staff** |  |  |
| **Technical** |  |  |
| **Operational** |  |  |

**Take note of the following:**

- Interactions among staff
- Interactions between staff and patient
- Stakeholder’s reactions/attitudes towards the VR
- How the VR intervention impacts clinical workflow
