# Supplementary material for: The use of virtual reality during medical procedures in a pediatric orthopedic setting: A mixed‐methods pilot feasibility study
Source: Paediatr Neonatal Pain. 2022 Apr 14;6(3):45–59. doi: 10.1002/pne2.12078 (PMC11514299; doi:10.1002/pne2.12078)
Supplement: Supplementary file 2 — Appendix S2 [file PNE2-6-45-s002.docx]

**Appendix S2**

**Focus Group Guide**

1. Can you tell us if your experience with VR? Have you ever played a VR game? Have you ever used VR in a clinical setting?

**VR PROVIDER:**

1. Can you tell us about your experience administering the VR intervention?
2. What did you find easy about using the VR system?
3. What did you find hard about using the VR system?
4. What did you find easy to understand about the VR system?
5. What did you find hard to understand about the VR system?

**HEALTHCARE PROVIDER:**

1. How did the VR intervention impact your work (performing the medical procedure)? Did it make it easier? Or harder?
2. Do you think the VR intervention helped your patient feel less pain or less anxious? (potential benefits and outcomes)
3. Would you use a VR intervention again with other patients? If so, for what types of medical procedures do you see VR being useful for?
4. Would you consider implementing VR as a distraction tool into your everyday practice? If so, what would be the best way to integrate VR into your practice? What resources would you need?

**EVERYONE:**

1. How could we improve the delivery of the VR intervention?
2. Do you value VR as an intervention? Are innovations practical in a hospital setting?
3. Any last comments or concerns?
