# Supplementary material for: The use of virtual reality during medical procedures in a pediatric orthopedic setting: A mixed‐methods pilot feasibility study
Source: Paediatr Neonatal Pain. 2022 Apr 14;6(3):45–59. doi: 10.1002/pne2.12078 (PMC11514299; doi:10.1002/pne2.12078)
Supplement: Supplementary file 3 — Appendix S3 [file PNE2-6-45-s001.docx]

**Appendix S3**

**Semi-Structured Interview Guide**

Date: _______/_______/_______ Participant Code: _______________

DD MM YYYY

1. Can you tell us if you have ever played with a VR game before? If so, in what context have you played a VR game? (For fun? During a medical procedure?) What was the VR game?
2. What did you find easy about using the VR game?
3. What did you find hard about using the VR game?
4. What did you find easy to understand about the VR game?
5. What did you find hard to understand about the VR game?
6. What did you like the most about using VR during your medical procedure?
7. What did you like the least about using VR during your medical procedure?
8. What would you change about your experience with VR?
9. Would you be willing to play a VR game again during another painful medical procedure?
10. Did the VR game help you forget about your medical procedure? Did it help you deal with the pain you were feeling or worries you had?
11. Would you recommend playing a VR game to someone else who needs to have a similar medical procedure as you?
12. Do you have any comments or concerns?
